# Supplementary material for: Epidemiologic, clinical, and therapeutic aspects of formally identified Echis romani bites in northern Cameroon
Source: PLoS Negl Trop Dis. 2025 Jul 28;19(7):e0013195. doi: 10.1371/journal.pntd.0013195 (PMC12303315; doi:10.1371/journal.pntd.0013195)
Supplement: S1 Appendix — The images/clip art appearing in the illustrated panels were hand-drawn by one of the authors (JPC) and are compatible with a CC BY 4.0 license. (DOCX) [file pntd.0013195.s001.docx]

**Appendix 1. Management Algorithm Recommended by Cameroonian Ministry of**

**Envenomation Patients (from 33; 34)**

**Gradation of edema:**

0. No edema.

1. Localized edema not exceeding the nearest joint.

2. Progressive edema not exceeding 2 contiguous joints.

3. Extensive edema not exceeding the root of the limb.

4. Edema extending beyond the root of the limb (hydrops).

**Gradation of bleeding:**

0. No bleeding.

1. Persistent local bleeding at fang marks for more than one hour.

2. Bleeding from the gums, nose, scars, and recent wounds.

3. Ecchymosis, hematoma, purpura, phlyctens.

4. Internal hemorrhage (peritoneal, meningeal, metrorrhagia, hematemesis, etc.).

**Gradation of neurological disorders:**

0. No neurological disorder.

1. Local anesthesia, tingling affecting the bitten limb.

2. Profuse sweat, saliva and vomiting, miosis.

3. Bilateral ptosis (±speech, vision, hearing and/or swallowing disorders).

4. Respiratory distress, impossibility to communicate.

**Gradation of whole-blood clotting time on dry tube (WBCT)**

1. Sample 2 mL blood in a clean dry glass tube
2. Let the tube stand without shaking it
3. Read the result after 20 minutes of waiting

**
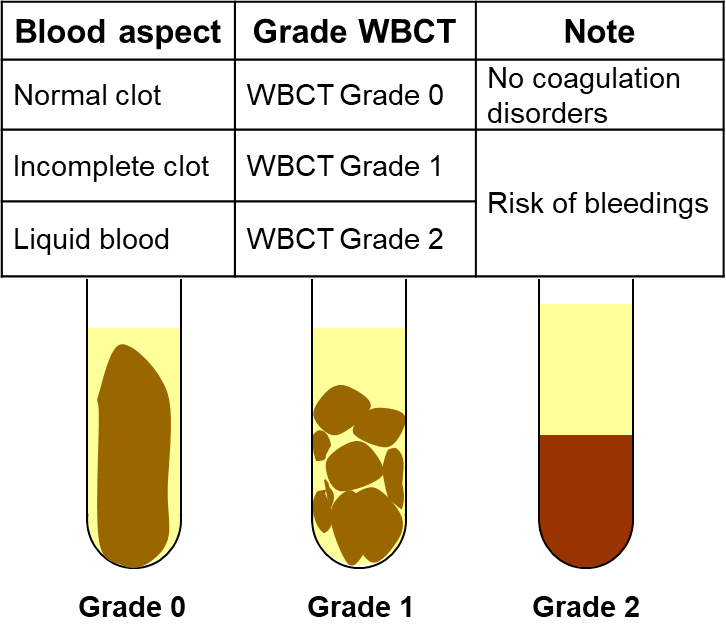
**
