# Supplementary material for: Epidemiologic, clinical, and therapeutic aspects of formally identified Echis romani bites in northern Cameroon
Source: PLoS Negl Trop Dis. 2025 Jul 28;19(7):e0013195. doi: 10.1371/journal.pntd.0013195 (PMC12303315; doi:10.1371/journal.pntd.0013195)

**Appendix 2. Management Algorithm Recommended by the Cameroon Ministry of Health for**

**Envenomated Patients (from 33; 34)**


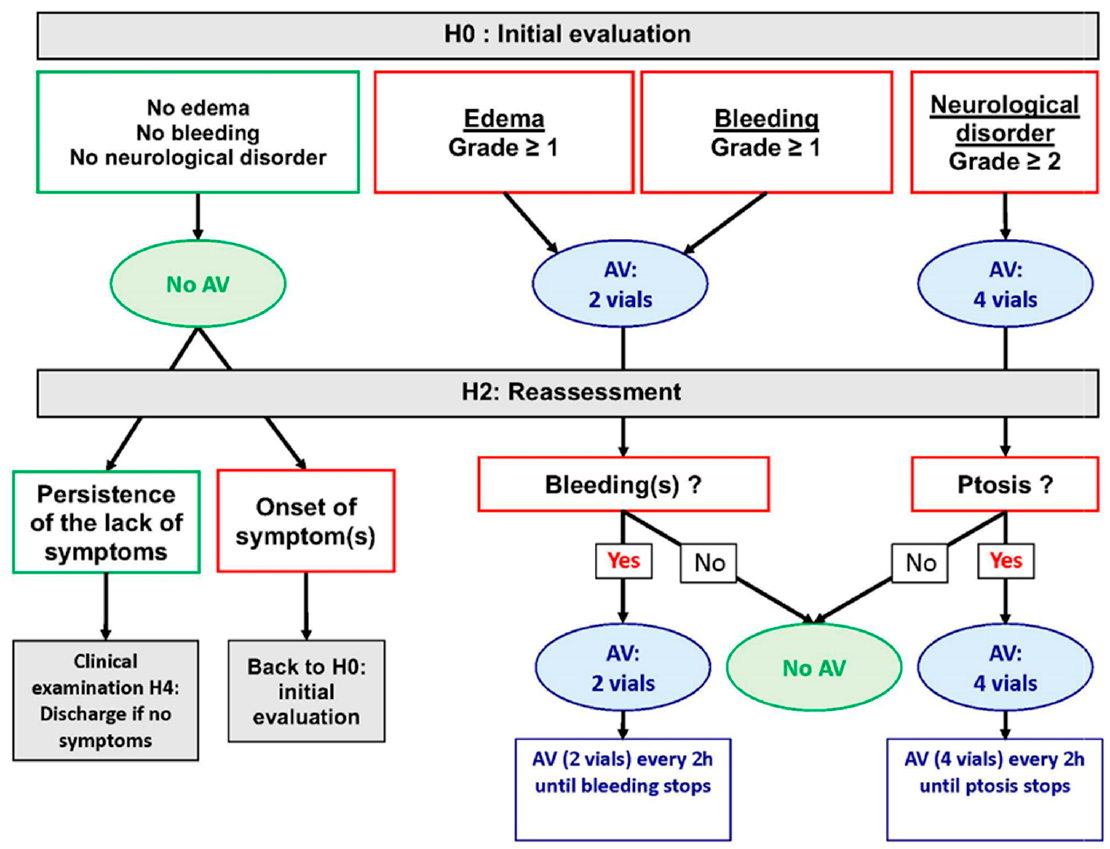

Supplement: S2 Appendix — (DOCX) [file pntd.0013195.s002.docx]
