# Supplementary material for: Epidemiologic, clinical, and therapeutic aspects of formally identified Echis romani bites in northern Cameroon
Source: PLoS Negl Trop Dis. 2025 Jul 28;19(7):e0013195. doi: 10.1371/journal.pntd.0013195 (PMC12303315; doi:10.1371/journal.pntd.0013195)
Supplement: Attachment — Submitted filename: Reviewers comments.docx [file pntd.0013195.s004.docx]

**Reviewers’ comments**

**Reviewer #1:**

The authors report the results of a clinical study aimed to characterize the symptoms and evolution of patients bitten by Echis romani.
The study was carried out on rural populations in northern Cameroon. Its strengths include formal identification of the snake responsible for envenomation and the standardized longitudinal follow-up of patients in several centres. The study is appropriately designed to assess the objectives, the population is well described and of sufficient size.
No concern about statistical analysis.
Ethical and regulatory requirements met.

Persented analysis match the plan. Results are clearly and completely presented and figures are of sufficient quality.

This well-documented clinical, biological and therapeutic study underlines the importance of Echis romani envenomation in the area covered by the study and the severity of the prognosis even after well-managed treatment with polyvalent antivenom serum.
Conclusions are well supported by the data. This helpful study opens the way to a better understanding of envenomation and its management and public health relevance.

The authors report the results of a clinical study aimed to characterize the symptoms and evolution of patients bitten by Echis romani.
The study was carried out on rural populations in northern Cameroon. Its strengths include formal identification of the snake responsible for envenomation and the standardized longitudinal follow-up of patients in several centres. The study is appropriately designed to assess the objectives, the population is well described and of sufficient size.
The manuscript is very well written.

This well-documented clinical, biological and therapeutic study underlines the importance of Echis romani envenomation in this area and the severity of the prognosis even after well-managed treatment with polyvalent antivenom serum.

Interestingly, the authors report delayed bleeding. These deserve to be explored by a new study in one or more centres with the possibility of carrying out complementary haemostasis examinations. These would include platelet counts to determine whether thrombocytopenia due to envenomation or associated with the administration of antivenom serum (such as anti-platelet factor 4 antibodies, as in HIT and VITT) could contribute to delayed/persistent bleeding. A dosage of plasma fibrinogen would also be of interest.

*We thank reviewer 1 for his positive feedback and comments.*

*We note with interest the suggestions made by the reviewer, which we consider to be particularly useful in significantly improving the protocol. We will certainly take them into account in future clinical trials.*

**Reviewer #2:**

This is an observational study, based on data collected in a study published elsewhere. Methods for data collection were sufficiently described herein and statistical analysis used simple chi-square tests. Ethical requirements were all met.

The description of the antivenom Inoserp PAN-AFRICA (IPA) did not include data of the venom-neutralizing efficacy tests (potency) of the product. It is known that the in vivo tests provide globally applicable standard metrics of antivenom efficacy, and results from these quality control tests need to be provided by the manufacturer as part of the batch release documentation. This information would be important to discuss the resolution of clinical manifestations of envenoming caused by Echis romani snakes.

*We thank reviewer 2 for his relevant comments.*

*However, Inoserp PAN-AFRICA is registered in Cameroon and many other countries. Its characteristics have been submitted to and accepted by the regulatory authorities. This information was also submitted to ESAA, the sponsor of the study, and to the Ethics Committees, which gave their approval. The potency of antivenom provided by the manufacturer meets WHO standards, i.e. one vial of antivenom neutralizes at least 250 LD_50_ of each species covered by the antivenom, including* Echis ocellatus. *We add this information to the text of the manuscript in lines 143-145:*

*« Each vial contains no more than 1 g of total proteins and neutralize at least 250 LD_50_ of the venom of* E. ocellatus*,* B. arietans*,* N. nigricollis *and* D. polylepis*. IPA from a single batch (#8IT11001; expiration date Nov. 2021) was used for all patients at all study centers. »*

The descriptive analysis showed the demographic characteristics and the clinical manifestations of patients envenomed. In this way, most of results are clearly presented, except for safety data on antivenom treatment, which was omitted. As allergic reactions are known to occur after administration equine derived immunoglobulins, both early and late reactions should have been presented in the study. Moreover, it was mentioned that the patients were evaluated approximately 15 days after hospitalization, to check for possible late reaction.

*As pointed out by the reviewer, the manuscript does not provide sufficient information on the safety data, which were detailed in a specific article whose results are briefly reported (lines 151-159). We have added references in the Patients and Methods section describing the procedure we used for data collection. We have added a paragraph on antivenom tolerance (lines 242-249):*

*“* *No serious adverse events (i.e., angioedema and bronchospasm) were reported in the 87 patients who received IPA and underwent clinical evaluation. No urticaria was observed, but 3 patients experienced pruritus (2 within 2 hours and 1 more than 2 hours after IPA administration), and another showed diffuse erythema within 2 hours after IPA administration.*

*“In terms of causality assessment, no patient experienced adverse events that were considered probably related to IPA administration according to the Naranjo algorithm (see Reference 34 for more methodological details). The three patients with pruritus and the patient with diffuse erythema were considered possibly related to IPA administration).”*

It is noteworthy that seven patients who were not bleeding on arrival at hospital, presented hemorrhage hours after admission and five who had recurrence of bleeding despite the administration of IPA. These 12 patients represent 14% of patients with clinical manifestations of envenomation. In this way, the therapeutic response of patients bitten by E. romani to the scheduled treatment with Inoserp PAN-AFRICA deserved a more detailed analysis.

The authors mentioned that the causes of prolonged bleeding episodes are uncertaint, and one possible cause for these findings include venom recirculation. In this way, would the recommended regimen of initial administration of 2 vials have been sufficient to reverse the hemorrhagic manifestations?

*The various hypotheses, including the recirculation of the antivenom after a period of normalization, could not be satisfactorily investigated in this study conducted under real life conditions and without systematic and detailed biological and hematological data. Treatment with a high dose of antivenom administered at the beginning of treatment was not included in the Cameroonian Ministry of Health protocol that we followed. In the context of a treatment that is fully paid for by the patient, such a procedure would be very costly for the patient and his family, who generally do not have the means to finance the treatment. We have added a sentence to answer the reviewer's question (lines 408-416):*

*"It is possible that the initial administration of more vials of antivenom would produce different results in the 15% of patients who experience delayed or recurrent bleeding. However, given the limited availability of antivenom, the protocol used in many countries in sub-Saharan Africa is a sequential approach, in which antivenom is administered in successive doses, with repetition based on clinical progress [70]. This approach therefore requires close monitoring of the patient with a clinical examination every 2 hours to re-administer the antivenom if the clinical presentation does not improve or worsens. However, it is uncertain whether administering a higher dose of antivenom is more effective, as the recirculation of the venom may be due to its sequestration in a part of the body inaccessible to the antivenom [56; 69]."*

Figure 6 and 7 are not essential, as data is sufficiently described in the Results section.

*We believe these two figures are useful because, on the one hand, they provide more detail than the text in the results section and, on the other hand, they visually illustrate the trends mentioned in the manuscript.*

**Reviewer #3:**

The study made a careful analysis of the cases of accidents caused by the snake Echis romani, with due ethical follow-up, in rural populations in northern Cameroon.
The antivenom Inoserp PAN-AFRICA proved effective in most treatments, but the persistence of symptoms and mortality could be better explored. Could it be associated with specificity, given the large number of venoms (14 species) used for preparation?
It may be beneficial to consider providing additional information regarding the characterisation of the antivenom, as this could further enrich the discussion.
It is my understanding that there are no problems with the statistics.

The results and figures are presented in a clear and detailed manner. It has been indicated accidents, with young rural workers, especially children and adolescents, being the most frequently affected. The seasonality of the accidents was assessed, with the highest incidence occurring at the end of the dry season and during the two rainy seasons. The majority of patients exhibited symptoms indicative of both poisoning and haemostasis disorders, manifesting as bleeding during their period of hospitalisation. The antivenom Inoserp™ PAN-AFRICA was administered in most cases, with positive outcomes. However, some patients suffered from prolonged bleeding, and the mortality rate remains significant. A more precise description of the serum might offer valuable insights for the discussion of this condition.

*We thank reviewer 3 for their insightful comments.*

*All available clinical and biological data were taken into account and used in the analysis of the cases. This was a real-world study with limited biological investigations due to the capacity of the health centers. We cannot provide a more detailed description of the cases to explain their progression and outcomes.*

*Regarding the specifications of Inoserp PAN-AFRICA, we have added the following sentences (lines 143-145):*

*« Each vial contains no more than 1 g of total proteins and neutralize at least 250 LD_50_ of the venom of* E. ocellatus*,* B. arietans*,* N. nigricollis *and* D. polylepis*. IPA from a single batch (#8IT11001; expiration date Nov. 2021) was used for all patients at all study centers.*

It is important to point out the severity of the haemorrhagic complications caused by Echis romani and the limited efficacy of antivenom in cases of prolonged or recurrent bleeding. Although antivenom has shown positive results in initially stopping bleeding, the persistence of haemorrhagic episodes and the high lethality indicate gaps in clinical management and in understanding the mechanisms involved. The conclusion could be more detailed in recommending improvements in treatment management, as well as investigating the causes of prolonged complications.

*The reviewer's suggestion is perfectly valid. We have added a sentence to the conclusion to follow this recommendation:*

*" Further studies are needed to better understand the causes of recurrent bleeding. Measuring the concentration and distribution of the venom in the body, particularly in the plasma, would help identify the causes of these recurrences and allow appropriate treatments to be proposed. Furthermore, if it is challenging to increase the number of initial vials, expanding the potency of the antivenom could improve outcomes at a reduced cost."*

Minor Revision. The production and characterisation of the serum could be improved through greater detail, in view of the cases of poisoning by Echis romani.

*This point has already been discussed.*
